# Supplementary figures and images for: A novel hybrid-based approach of snort automatic rule generator and security event correlation (SARG-SEC)
Source: PeerJ Comput Sci. 2022 Mar 2;8:e900. doi: 10.7717/peerj-cs.900 (PMC9044335; doi:10.7717/peerj-cs.900)

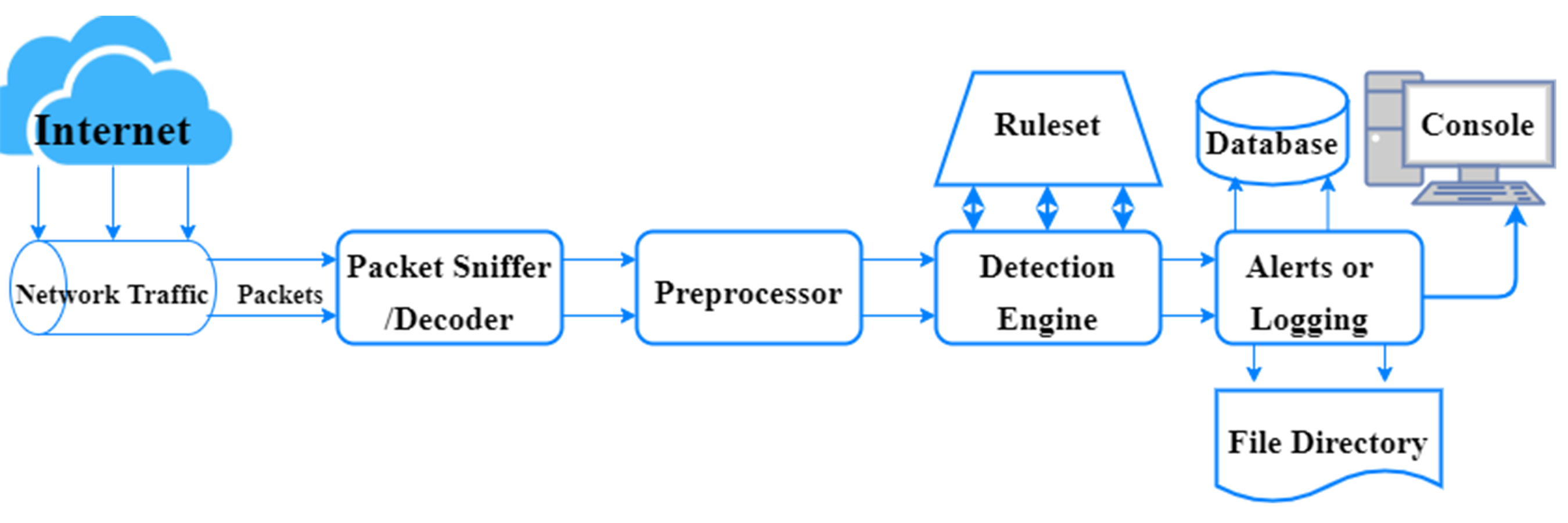

Supplement: Supplemental Information 1 [file peerj-cs-08-900-s001.png]

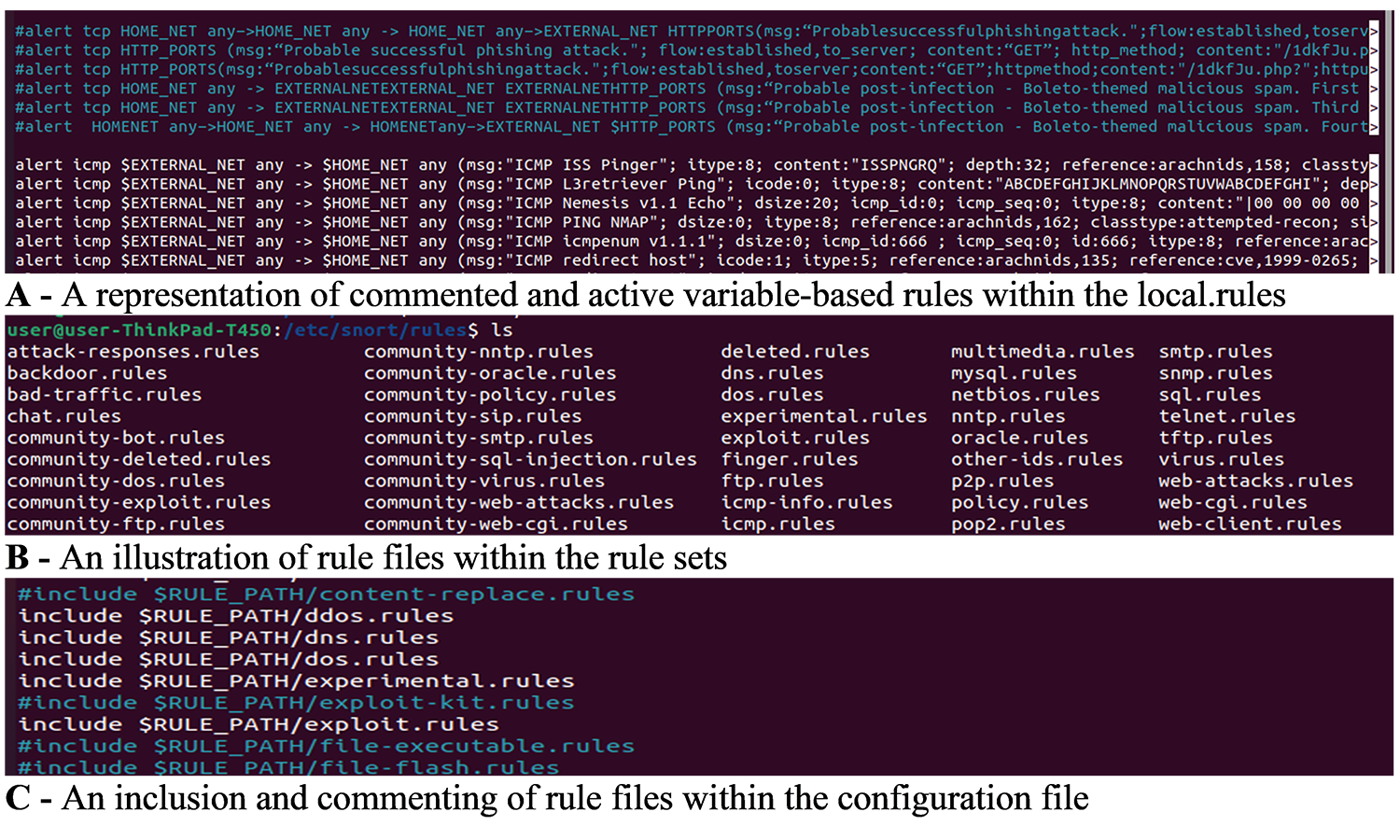

Supplement: Supplemental Information 2 — (A) A representation of commented and active variable-based rules within the local.rules. (B) An illustration of rule files within the rule sets. (C) An inclusion and commenting of rule files within the configuration file. [file peerj-cs-08-900-s002.png]

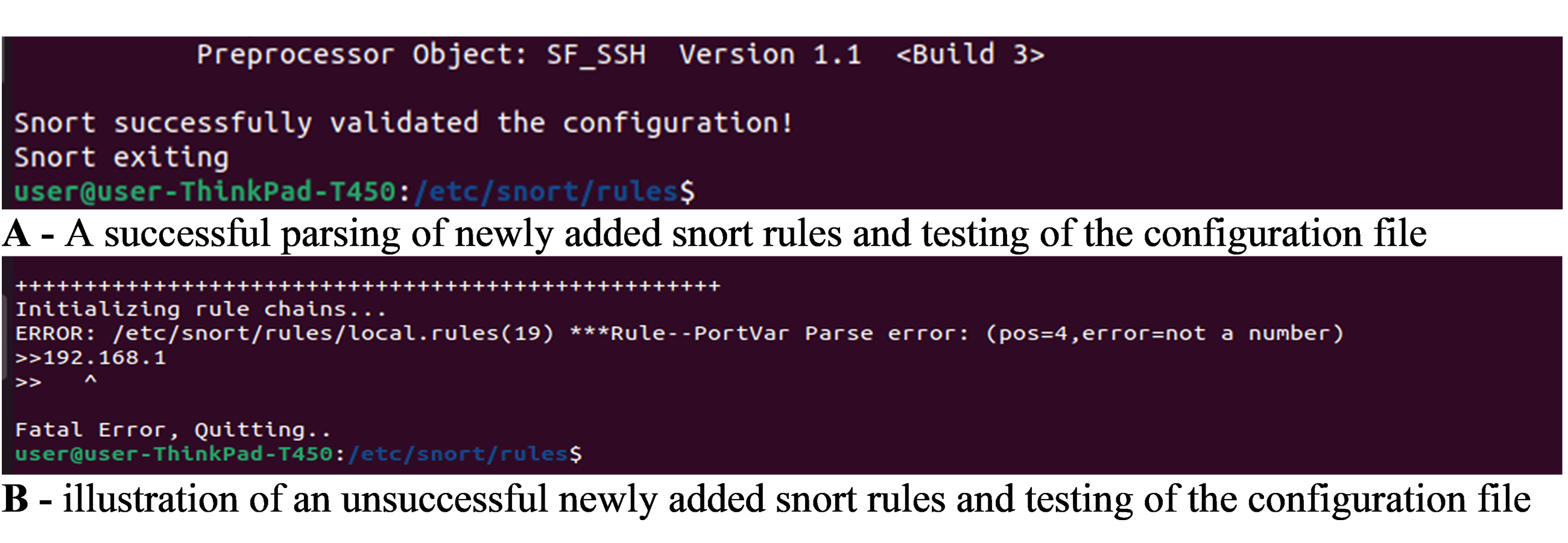

Supplement: Supplemental Information 3 — (A) A successful parsing of newly added Snort rules and testing of the configuration file. (B) Illustration of an unsuccessful newly added Snort rules and testing of the configuration file. [file peerj-cs-08-900-s003.png]
